# Detection of SARS-CoV-2 infection prevalence in 860 cancer patients with a combined screening procedure including triage, molecular nasopharyngeal swabs and rapid serological test. A report from the first epidemic wave

**DOI:** 10.1371/journal.pone.0262784

**Published:** 2022-02-02

**Authors:** Anna Candoni, Giuseppe Petruzzellis, Alessandra Sperotto, Victoria Andreotti, Marco Giavarra, Carla Corvaja, Alessandro Minisini, Chiara Comuzzi, Carlo Tascini, Renato Fanin, Gianpiero Fasola

**Affiliations:** 1 Department of Hematology and SCT, Santa Maria della Misericordia Hospital, ASUFC, Udine, Italy; 2 Department of Oncology, Santa Maria della Misericordia Hospital, ASUFC, Udine, Italy; 3 Department of Infectious Diseases, Santa Maria della Misericordia Hospital, ASUFC, Udine, Italy; 4 DAME, University of Udine, Udine, Italy; Rutgers University, UNITED STATES

## Abstract

**Introduction:**

Even if now we have available the weapon of vaccination against SARS-CoV-2, the patients with cancer remains a very frail population in which frequently the immunologic response to vaccination may be impaired. In this setting, the SARS-CoV-2 infection screening retains a great value. However, there are still limited data on the feasibility and efficacy of combined screening procedures to assess the prevalence of SARS-CoV-2 infection (including asymptomatic cases) in cancer outpatients undergoing antineoplastic therapy.

**Patients and results:**

From May 1, 2020, to June 15, 2020, during the first wave of SARS-CoV-2 pandemic, 860 consecutive patients, undergoing active anticancer therapy, were evaluated and tested for SARS-CoV-2 with a combined screening procedure, including a self-report questionnaire, a molecular nasopharyngeal swab (NPS) and a rapid serological immunoassay (for anti-SARS-CoV-2 IgG/IgM antibodies). The primary endpoint of the study was to estimate the prevalence of SARS-CoV-2 infection (including asymptomatic cases) in consecutive and unselected cancer outpatients by a combined screening modality. A total of 2955 SARS-CoV-2 NPS and 860 serological tests, in 475 patients with hematologic cancers and in 386 with solid tumors, were performed. A total of 112 (13%) patients self-reported symptoms potentially COVID-19 related. In 1/860 cases (< 1%) SARS-CoV-2 NPS was positive and in 14 cases (1.62%) the specific serological test was positive (overall prevalence of SARS-CoV-2 infection 1.62%). Of the 112 cases who declared symptoms potentially COVID-19-related, only 2.7% (3/112) were found SARS-CoV-2 positive.

**Conclusions:**

This is the largest study reporting the feasibility of a combined screening procedure (including triage, NPS and serologic test) to evaluate the prevalence of SARS-CoV-2 infection in cancer patients receiving active therapy, during the first epidemic wave and under the restrictive lockdown measures, in one of the active areas of the SARS-CoV-2 circulation. Lacking specific recommendations for the detection of asymptomatic SARS-CoV-2 cases, a combined diagnostic screening might be more effective to detect the exact prevalence of SARS-CoV-2 in neoplastic patient population. The prevalence can obviously change according to the territorial context, the entity of the restrictive measures adopted and the phase of the epidemic curve. However, its exact and real-time knowledge could be important to balance risks/benefits of oncologic treatments, avoiding (if the prevalence is low) the reduction of dose intensity or the selection of less intensive (but also less effective) anti-cancer therapies.

## 1. Introduction

SARS-CoV-2 infection and related disease (COVID-19) has been an ongoing global health emergency since early 2020 [1–3]. The epidemic in Europe reached its first peak in March 2020 and, unfortunately, the European Community has been involved in further, and even more intense, epidemic waves with over 51 million confirmed cases of infection as of May 2021 and over 1 million related deaths (over 125.000 related deaths only in Italy).

Epidemiological, clinical, and therapeutic knowledge on this disease is still partial and in rapid update and there are still few epidemiological data on its real prevalence (including symptomatic and asymptomatic cases), but it has become increasingly evident that the role of asymptomatic carriers is very important for the infection spreading and for maintenance of a human viral reservoir [4–7]. Furthermore, the worldwide emergence of SARS-CoV-2 variants is a further cause of concern and need to be monitored closely [8]. Even if we have now available the vaccination option against SARS-CoV-2, the patients with cancer remains a very frail population in which the efficacy of vaccination may be unsatisfactory with a higher risk for a severe COVID-19. In this scenario the management of cancer patients underling antineoplastic therapy remains very challenging [9–15]. Furthermore, in patients with solid and hematological tumors, a clinical anamnestic screening by specific triage alone may not be appropriate to intercept SARS-CoV-2 infection cases since some of the symptoms of malignancy may be similar as those of SARS-CoV-2 infection [16, 17]. For this reason, it is important, in cancer populations, especially in the outpatient setting, to define the most effective and targeted monitoring strategies in order to detect, as accurately as possible, the prevalence of SARS-CoV-2 infection in a definite temporal context and geographical area.

Herein, we report the feasibility and efficacy of a combined triple screening strategy (including triage, nasopharyngeal swabs and serological test) to detect the SARS-CoV-2 infection prevalence, in a large cohort of cancer patients undergoing active antineoplastic therapy between May and June 2020.

## 2. Patients and methods

This is a prospective cohort study including 860 consecutive outpatients with solid cancer or hematological malignancies treated at the University Hospital of Udine-ASUFC, Italy, during the first wave of COVID-19 pandemic.

The primary goal was to assess the prevalence of SARS-CoV-2 infection (symptomatic or asymptomatic cases) using a combined triple screening strategy (including triage, nasopharyngeal swabs and serological test), in patients with active cancer requiring antineoplastic therapy, between May 01 and June 15, 2020. The choice of this period of epidemiological analysis was performed taking into account that it was a temporal phase of active circulation of SARS-CoV-2 in Italy and that, given the maximum incidence of the infection recorded in March 2020, there was an appropriate time frame for the development of an antibody response in potentially exposed cases that were tested.

The study protocol was approved by the Ethics Committee of Friuli Venezia Giulia Region-IT (N° CERU FVG-2020-Os-187) and was conducted in accordance with the Declaration of Helsinki.

The criteria for inclusion in the study were: 1) diagnosis of malignancy under active anticancer therapy; 2) access to the onco-hematologic outpatient department, from May 01, 2020, to June 15, 2020, with a completion of a self-reported triage questionnaire; 3) performance of at least one molecular naso-pharyngeal swab (NPS) and one rapid serologic test for SARS-CoV-2 during the study period; 4) age > 18 years; 5) signature of written informed consent.

The collected and analyzed information’s included: specific patient biographical data, type of cancer (by site), stage of neoplasm (advanced vs. early), type of therapy (conventional chemotherapy, immunotherapy, target therapy), line of therapy (first line, salvage, palliative), potentially COVID-19 related symptoms (fever, sore throat, cough or dyspnea, ageusia, anosmia, headache, pharyngitis, diarrhea, nausea, vomiting), the number of SARS-CoV-2 molecular NPS performed in the study period, the percentage of positive SARS-CoV-2 NPS, the number of rapid serological tests performed and percentage of positive.

### 2.1 Prevention and social distancing measures adopted during the study period

The epidemiological analysis was performed during a first lockdown period (first epidemic wave) in which schools in Italy were closed to in-person activity, the work activities were reduced, and remote work was encouraged. Circulation was permitted with a surgical mask; access to store and bar activities was restricted. All patients with active cancer were recommended to reduce as much as possible extra-family contacts. Access to the day hospital department was allowed only with surgical mask, and after completion of a triage questionnaire. All healthcare workers carried out their activities with personal protective equipment (PPE) and were also tested for SARS-CoV-2 with an RT-PCR NPS every two weeks, by active surveillance.

### 2.2 Questions included in the Day Hospital Pre-Access Triage procedure

The triage procedure consisted of a self-report questionnaire including the following key questions: (1) “Have you had fever ≥37°C in the last 14 days?” (2) “Have you had any of the following symptoms in the last 14 days: sore throat, cough or breathing difficulty, loss of taste and smell?” (3) “Have you been in close contact with confirmed SARS-CoV-2 infected persons in the last 14 days?” (4) “Have you been asked to self-quarantine and/or have you (or one of your family members) been tested positive for the SARS-CoV-2 virus?”. The triage questionnaire was considered positive if the patient reported at least one positive answer. Besides, patients received concurrent measurement of body temperature (BT) and were asked to declare the results of any previous SARS-CoV-2 test. All positively triaged patients underwent the same day molecular NPS control and returned home until the response of the swab test was available (generally available within 12–24 h).

### 2.3 Molecular nasopharyngeal swab (NPS) and Serological Test

The RT-PCR NPS was performed by dedicated and trained nursing staff, at least once in all 860 patients and before each access to the day hospital department, regardless of the triage result; it was additionally performed in all cases of self-reported potentially COVID-19 related symptoms. In addition to the molecular NPS, all patients were tested with one rapid qualitative serological test, in order to intercept, as extensively as possible, patients with a previous contact with the SARS-CoV-2 virus. To assess the presence of anti-SARS-CoV-2 antibodies we used a qualitative commercially available point-of-care lateral flow chromatographic immunoassay (Cellex qSARS-CoV-2 IgG/IgM cassette Rapid Test, Cellex, Inc., NC, USA) that can simultaneously detect IgM and IgG antibodies against SARS-CoV-2 in human blood, with a reported overall sensitivity of 98,4% and specificity of 96,4%.

### 2.4 Statistical analysis

Basic descriptive statistics were used to analyze and report patients’ characteristics. Quantitative variables are described as the mean±standard deviation or median and range, whereas qualitative variables were described as number and percentages. Differences in categorical variables were analyzed using the Fisher exact test. The chi-square test was used to analyze proportions when appropriate. All tests were performed two-sided at a significance level of 0.05. The small number of events precludes multivariate analysis. Statistical and graphical analyses were performed using MedCalc (version 19.2.1).

## 3. Results

### 3.1 Characteristics of the tested patients and of symptomatic cases

Eight hundred and sixty patients with malignancy were tested, of whom 474/860 (55%) had hematologic malignancies and 386/860 (45%) had solid tumors (**Table 1**). The most frequent neoplasms were lymphomas in 198/860 (23%) cases, breast cancer in 103/860 (12%) cases, multiple myeloma in 103/860 (12%) cases, acute leukemia in 83/860 (9.5%) cases, and lung cancer in 81/860 (9%) cases. The median age of patients was 64 years (range 17–91). As reported in **Table 1**, 41% (356/860) of patients had newly diagnosed cancer while 59% (504/860) had a relapsed or advanced/refractory neoplastic disease. All patients were receiving an active anticancer therapy and specifically: 327/860 (38%) chemotherapy alone, 373/860 (43%) immunotherapy (in 185 cases alone and in 188 cases in combination), 122/860 (14%) target therapy±immunotherapy, and 49/860 (6%) other supportive/palliative treatments. None of these patients had received anti SARS-CoV-2 vaccination.

**Table 1:** Patients’characteristics.

Solid Cancers	N°(%)	Hematologic Cancers	N°(%)
**Patients**	**386**	**Patients**	**474**
**Sex (Male/Female)**	**166/220**	**Sex (Male/Female)**	**265/209**
**Median Age (range)**	**64 (31–85)**	**Median Age (range)**	**63 (17–91)**
**Type of Disease** • Breast cancer • Lung cancer • Colorectal cancer • Pancreatic cancer • Gastric cancer • Skin cancer and Melanoma • Ovarian cancer • Head and neck cancer • Prostatic cancer • Renal and Urothelial cancer • Gynecological cancer (ovarian excluded) • Biliary tract cancer • Endocrine cancer • Other°	**103** **81** **54** **34** **24** **22** **16** **8** **9** **15** **10** **3** **2** **5**	**Type of Disease** • Lymphoma • Non-Hodgkin • Hodgkin • Cronic Lymphocytic leukemia • Acute Leukemia • Myelodysplastic syndrome • Multiple Myeloma • Myeloproliferative neoplasms • Other**	**198** **152** **46** **59** **83** **6** **103** **14** **11**
**Status of Disease** • First Diagnosis • Relapsed/Refractory disease	**98** **288**	**Status of Disease** • First Diagnosis • Relapsed/Refractory disease	**258** **216**
**Ongoing Therapy** • Chemotherapy alone • Chemotherapy ±Immunotherapy±TT • Immunotherapy alone • Target Therapy (TT) alone • Immunotherapy + TT	**186** **73** **84** **35** **8**	**Ongoing Therapy** • Chemotherapy alone • Chemotherapy ±Immunotherapy±TT • Immunotherapy alone • Target Therapy (TT) alone • Immunotherapy + TT • Other^^	**141** **104** **101** **76** **3** **49**

° 1 esophageal cancer, 1 peritoneal cancer, 1 testicular cancer, 1 tymoma, 1 glioblastoma

** 4 amyloidosis; 4 myelodysplastic syndrome+emoglobinuria; 3 hystiocytosis

^^ mainly steroids ± radiotherapy.

**Fig 1:**
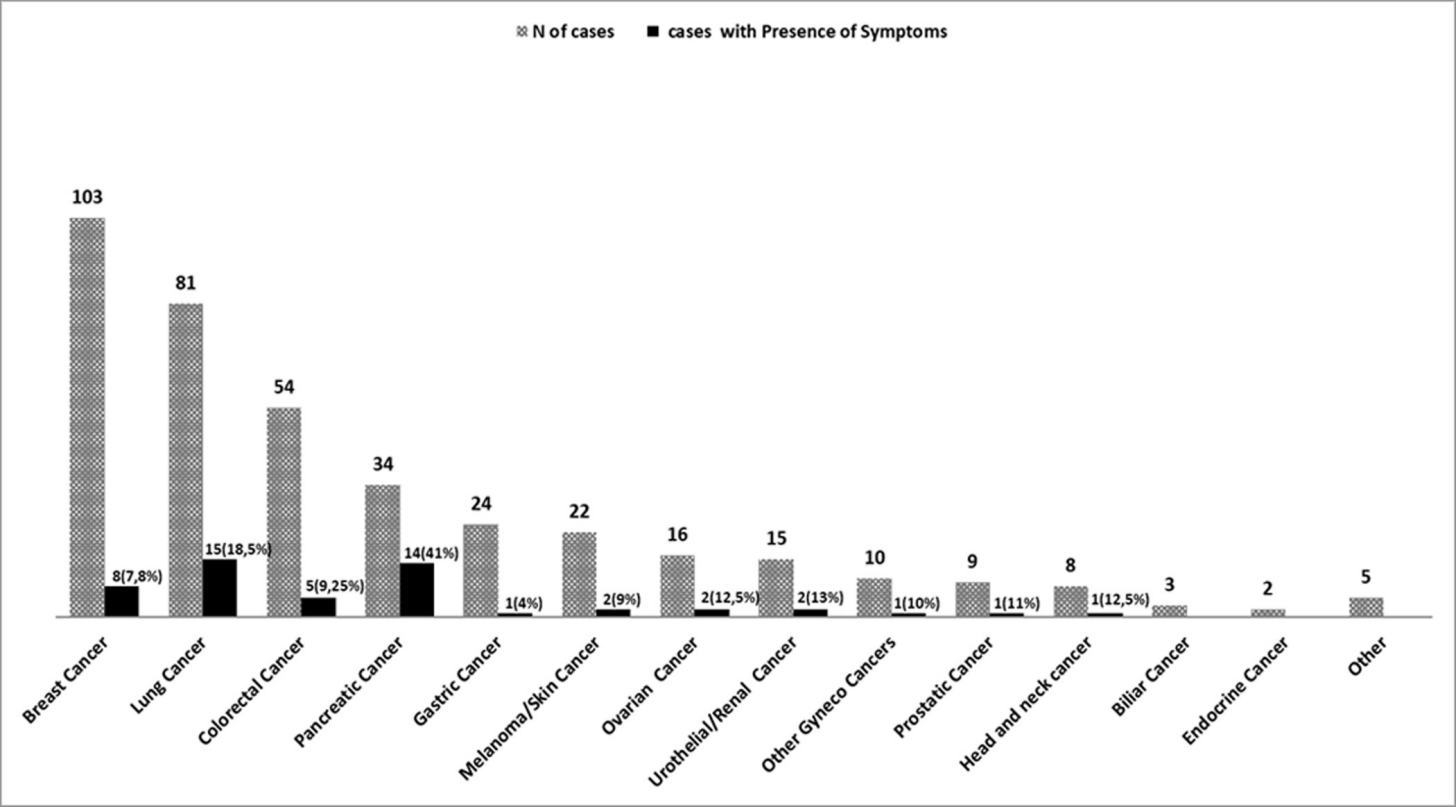
Number of cases according to the solid cancer type and percentage of patients who declared one or more symptoms potentially COVID-19-related at the specific triage procedure.

**Fig 2:**
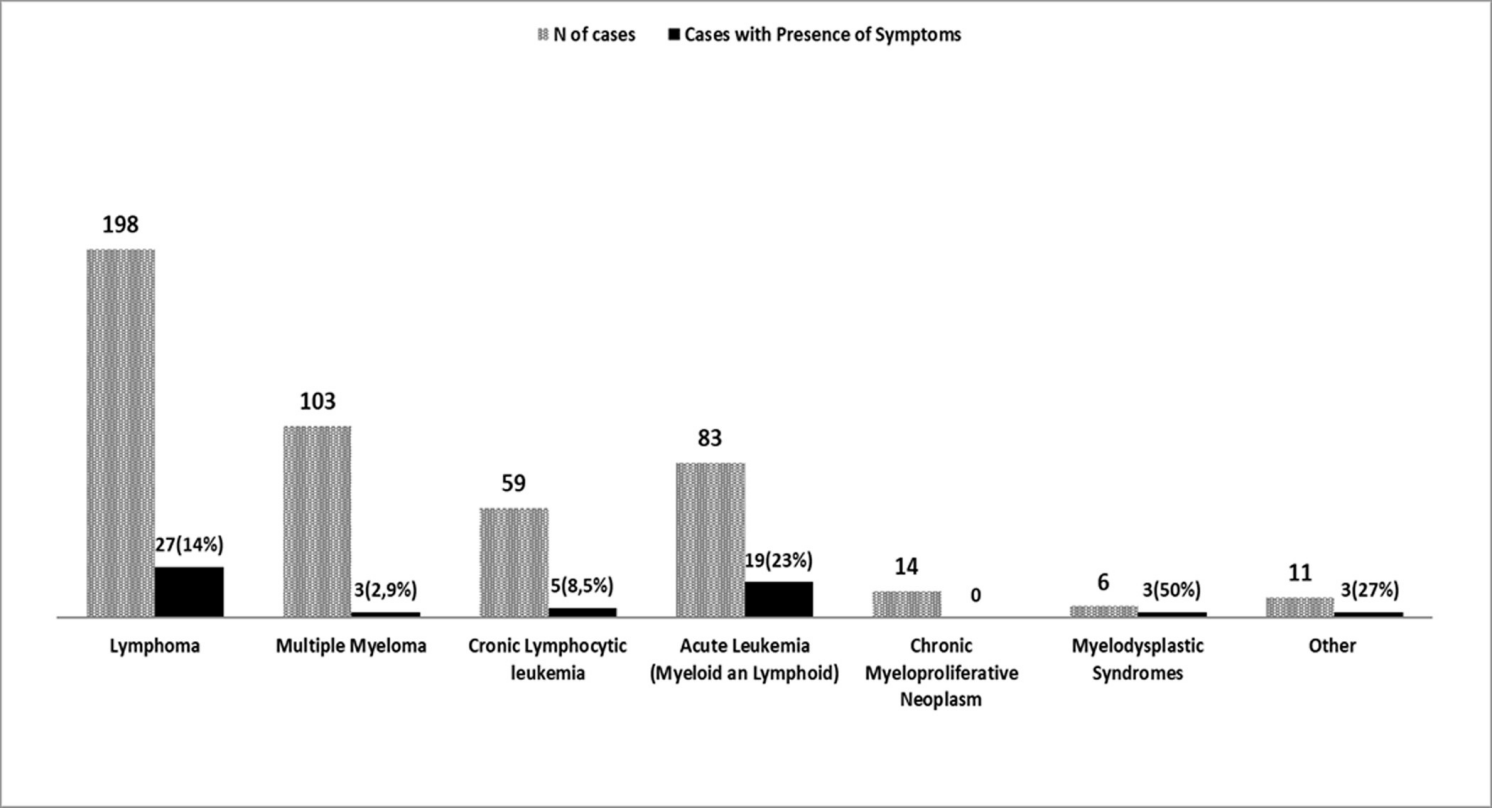
Number of cases according to the hematologic cancer type and percentage of patients who declared one or more symptoms potentially COVID-19-related at the specific triage procedure.

Of the 860 tested patients, 13% (112/860) declared one or more symptoms potentially COVID-19-related at the specific triage procedure performed at each day hospital access during the study period, without significant differences between patients with solid tumors and those with hematologic malignancies (13.5% vs. 13%). The most frequent declared symptom was fever > 37°C, that was reported in 7% (27/386) of patients with solid tumors and in 9,3% (44/474) of cases with hematological neoplasms. Among solid tumors, the presence of clinical symptoms (particularly the presence of fever > 37°C) was more relevant in patients with pancreatic (symptoms in 41% of cases) and lung cancers (symptoms in 18.5% of cases), as shown in **Fig 1**. In patients with hematological neoplasms, the presence of clinical symptoms (mainly fever > 37°C) was more frequent in patients with acute leukemia (23%), myelodysplastic syndromes (50%) and lymphomas (14%)-**Fig 2.**

### 3.2 Molecular NPS and rapid serological tests and results

As shown in **Table 2**, during the study period (6 weeks), 2955 molecular NPS were performed with a median of 4 NPS/patient (range, 1–9) and oncological patients underwent a higher number of RT-PCR NPS than hematological population (p<0.05). We found only 1 positive NPS/2955 (< 1%) in a patient with solid cancer (gastric carcinoma) symptomatic for fever. Therefore, a positive predictive value (PPV) of 0,89% (95% CI: 0,87–0.91%) was detected.

**Table 2:** Results of the combined screening procedure.

	TOTAL CASES	Solid Cancers	Hematologic Cancers
	860	386 (45%)	474 (55%)
Symptoms potentially SARS-COV-2 related ^1^			
**Present**	112 (13%)	52 (13,5%)	60 (13%)
**Absent**	748 (87%)	334 (86,5%)	414 (87%)
Nasopharyngeal Swabs (NPS) SARS-COV-2			
**Total number**	2955	1692	1263
**Median NPS/patient**	4 (1–9)	4 (1–9)	3 (1–9)
**Pt Positive**	1 (<1%)	1 (<1%)	0 (0%)
SARS-COV-2 IgG/IgM Rapid Test			
**Total Number**	860	386	474
**Positive**	14 (1,62%)	6 (1,55%)	8 (1,7%)
**IgG and IgM positive**	7	3	4
**Only IgG positive**	1	1	0
**Only IgM positive**	4	2	4

^1^ Clinical Manifestations that could be attributed to SARS-CoV-2 disease: fever, cough, dyspnea, fatigue, headache, myalgia, vomiting, diarrhea and neurological manifestations (anosmia, ageusia).

During the same period, 860 rapid serologic tests were performed (1 test each patient) and 14/860 (1.62%) were positive without significant differences between hematologic (8/474 positive-1.7%) and solid cancers (6/386 positive-1.55%) (p = 0,88). The overall seroprevalence of anti-SARS-CoV-2 antibodies (IgG and/or IgM) was 1.62%. Of the 14 cases with positive serologic test, IgG and IgM positivity was found in 7/14 (50%), IgG positivity only in 1/14 (7%), and IgM positivity only in 6/14 (43%) (**Table 2**). Of the 112 cases that declared potentially COVID-19-related symptoms at triage, only 2.7% (3/112) resulted SARS-CoV-2 positive (1 NPS + serologic test positive; 2 with only serologic test positive).

### 3.3 Characteristics of SARS-CoV-2 positive cases

Features of the 14 SARS-CoV-2 positive cases are reported in **S1 Table**. Of them, 8 cases had hematologic malignancies (4 non-hodgkin’s lymphomas, 3 multiple myeloma and 1 myelodysplastic syndrome) and 6 cases had solid tumors (2 gastric cancer, 1 colon cancer, 1 lung cancer, 1 pharyngeal cancer and 1 breast cancer). At the triage procedure 3/14 positive cases (21%) declared symptoms (1 fever, 1 cough, 1 fever and diarrhea) while 11/14 of SARS-CoV-2 positive cases (79%) were completely asymptomatic both at the time of testing, during all the study period and in the 2 months before the study starts.

Overall, using a combination of the 2 tests (NPS and serological test), we found an overall prevalence of SARS-CoV-2 infection, in the analyzed population, of 1.62% (14 positive cases /860 cases tested) with a prevalence of symptomatic cases < 1% (3 cases/860) and a prevalence of asymptomatic cases of 1,27% (11 cases/860).

No secondary SARS-CoV-2 infections were detected among healthcare workers who were in close proximity to these patients.

## 4. Discussion

Since the start of SARS-CoV-2 epidemic, it soon became clear that patients with cancer represent a very vulnerable population with a high risk of severe COVID-19 and a high mortality rate [12, 13, 17–21]. For this reason, both the oncological and hematological societies recommend constant and careful screening to identify and isolate SARS-CoV-2 infected patients, avoiding infection spreading among this very frail population [22–25]. However, in patients with cancer, very limited real-world data are available regarding the efficacy of combined screening procedures to detect the prevalence of SARS-CoV-2 infection [17, 26, 27].

In this prospective study, we analyzed a large cohort of cancer patients treated, at the University Hospital of Udine (Italy), during the first wave of COVID-19 pandemic, in order to assess, using a combined screening procedure, the effective prevalence of SARS-CoV-2 infection (including symptomatic and asymptomatic cases). Of note, in our geographic area (Friuli-Venezia Giulia Region; North-East of Italy), during our study time-lapse, the cumulative incidence of SARS CoV-2 infection was 235 per 100.000 people as of May 1^st^,2020, and 280 per 100.000 as of June 15^th^, 2020 [16]. During the six weeks of surveillance, 860 patients with cancer, undergoing active treatment, were strictly monitored with a questionnaire-triage procedure, periodic RT-NPS and a serological test. Our findings suggest that a questionnaire-based triage system, even if accurate and important, has a low positive-predictive value (0,89%; 95% CI: 0,87–0,91%) for the identification of cancer patients with SARS-CoV-2 infection since a differential diagnosis between tumor-related symptoms (fever for leukemia or paraneoplastic syndromes, dyspnea for lung cancer) or treatment-related symptoms (diarrhea and dysgeusia for mucositis, fever for gemcitabine) and COVID-19-related symptoms is always very difficult. In fact, of the 112 patients whose reported potentially COVID-19-related symptoms at triage, only 2.7% (3/112) were actually SARS-CoV-2 positive. This data highlights, as we have recently reported, the opportunity of a triage screening implementation in the onco-hematological setting to avoid unnecessary treatment delays [16, 27, 28]. We also underscore that 11/14 (79%) of our cases, in which a contact with SARS-COV-2 was confirmed, had an asymptomatic course of infection that was documented only by a detection of the specific antibody production. In view of that, a combined screening modality, beyond a single symptom-driven approach, would be very useful to better identify the SARS-CoV-2 prevalence in cancer patients overcoming the possible limitations of a single procedure.

Obviously, the current gold standard confirming SARS-CoV-2 infection remains, as recommended by the Center for Disease Control, the collection of nasopharyngeal swabs followed by SARS-CoV-2 RNA detection using reverse-transcriptase PCR (RT-PCR). However, the results of the molecular NPS test might be affected by stage of infection and/or quality of the sample and the sensitivity and specificity for RT-PCR NPS are around 70% and 95%, mostly evaluated in symptomatic patients [29–31]. Some studies showed a low concentration of viral RNA in samples from asymptomatic patients; therefore, the real sensitivity of NPS in asymptomatic carriers could probably be lower than in the symptomatic cases and also false-negative NPS test results have been reported early in the course of SARS-CoV-2 infection [32–36]. Regarding the serological tests detecting IgG and IgM antibodies against SARS-CoV-2 they might be useful in neoplastic population [17, 18, 37, 38]. They are easy to perform, could intercept previous asymptomatic infections and could help assessing the immune status of the patient. However, serological tests (quantitative or qualitative), if used alone, as a screening procedure, may have various weaknesses. In fact, it is well known that the antibody production may be impaired in immuno-compromised host, resulting in a lower protection against this infection and also a lower response to the vaccination. As a consequence, the possibility of false negative serology test cannot be excluded, resulting in an underestimated SARS-COV-2 prevalence in this setting [39–41]. The declared sensitivity and specificity of the rapid serological tests we used (Cellex qSARS-CoV-2 IgG/IgM Rapid Test) is 93.75% and 96.40%, respectively. Therefore, considering that we performed 860 rapid tests, it has to be considered both the false-positive (especially in only IgM positive cases) and false negative results [38]. Surprisingly, in a SARS-CoV-2 antibody seroprevalence study, performed by Cabezon-Gutierrez et al., high prevalence of IgG/IgM antibodies was detected in a relevant proportion of cancer patients (31,4%, 72/229 patients), mostly asymptomatic [42]. The probability of SARS-CoV-2 seropositivity wasn’t influenced by sex, type of treatment and cancer stage, whereas was significantly higher in cancer patients with pneumonia. However, in the Spanish region where the cancer center is located, a total cumulative incidence of 835 cases per 100,000 inhabitants and a prevalence of IgG in the general population of 20.2% was found, which could possibly explain this high IgG prevalence recorded in the cancer population [42]. Conversely, in a recent French study the reported SARS-CoV-2 seroprevalence under the first epidemic wave, in a large cancer population (1011 cases), was very low (1,7%) [17]. These results have recently been confirmed in an Italian study (performed in the Marche region) in which the SARS-CoV-2 seroprevalence in 949 cancer patients undergoing treatment was 0,7% [43]. Also in our study, according our geographic context, we observed a low prevalence (1,62%) of SARS-CoV-2 infection in a cancer patient population undergoing active therapy. These results suggest that the majority of our cancer patients remained uninfected during the first wave of SARS-CoV-2 pandemic, despite the active circulation of SARS-CoV-2 in our geographic area. Although this is a very good situation, indicating the efficacy of restrictive measures adopted, at the other side of the coin, it means that the neoplastic population in our area, during the study period, was immunologically naive to SARS-CoV-2 and not protected from a subsequent epidemic wave. This scenario suggested us to confirm the restrictive measures even after the peak of the first epidemic wave to maintain the therapeutic area of day hospital virus free.

We are aware that this study has some important limitations. The regional incidence of SARS-CoV-2 infection during the study period was not particularly high (around 250–280 cases/100.000 habitants) and there are no available data on concomitant seroprevalence in the general population in our geographic area (North-East of Italy) to compare the prevalence of our cancer population with that of the general population. In addition, our combined screening strategy is quite expensive and could probably be adopted only in a phase of epidemic expansion. Moreover, this study encapsulates and records what happened in mild 2020. However, it remains one of the largest studies evaluating the efficacy of combined screening procedures and prevalence of SARS-CoV-2 infection (including asymptomatic cases) in a cohort of cancer patients receiving anti-cancer treatment, during the first epidemic peak, in one of the areas of the SARS-CoV-2 active circulation. Lacking specific recommendations for the detection of asymptomatic SARS-CoV-2 cases, a coupled screening approach (triage, NPS, serological test) could be useful in improving the detection of SARS-Cov-2 infection prevalence in neoplastic patient populations, including the silent infection cases. Obviously, the prevalence data can be different according to the territorial context, to the entity of the restrictive measures adopted and also to the epidemic curve. Its knowledge is important to balance risks/benefits of oncologic treatments and to avoid, if the prevalence is low, the reduction of dose intensity or the selection of less intensive, but also less effective, anticancer therapies [1, 44].

In the coming months, we should remain cautious and, in this unstable pandemic context, a combined screening procedure could be promptly readopted, to improve the control of virus transmission, in case of additional waves of this highly dangerous infectious disease, particularly in countries or context with a low rate of vaccinated population.

## Supporting information

S1 Table Characteristics of the 14 SARS-CoV-2 positive cancer patients. *No symptoms during the study period nor in the 3 months before.
(DOCX)
