# Supplementary material for: Detection of SARS-CoV-2 infection prevalence in 860 cancer patients with a combined screening procedure including triage, molecular nasopharyngeal swabs and rapid serological test. A report from the first epidemic wave
Source: PLoS One. 2022 Feb 2;17(2):e0262784. doi: 10.1371/journal.pone.0262784 (PMC8809545; doi:10.1371/journal.pone.0262784)
Supplement: S1 Table — *No symptoms during the study period nor in the 3 months before. (DOCX) [file pone.0262784.s001.docx]

**S1 TABLE.** **Characteristics of the 14 SARS-CoV-2 positive cancer patients.**

| **ID Patient** | **Sex** | **Age** | **Disease** | **Ongoing Therapy** | **Regimen** | **Symptoms** | **N° of NPS** | **Concomitant**  **NPS** | **SARS-COV-2 IgG/IgM**  **Rapid Test** | **Positive Triage (at least one)** | **Infectious disease specialist**  **Evaluation** |
| --- | --- | --- | --- | --- | --- | --- | --- | --- | --- | --- | --- |
| 1 | **F** | **69** | **Multiple Myeloma** | **Chemotherapy** | **Velcade,Thalidomide, Desametazone** | **No*** | **4** | **Yes (negative)** | **Weak IgM** | **No** | **Yes** |
| 2 | **M** | **78** | **Myelodysplastic Syndrome (MDS)** | **Chemotherapy** | **Azacitdine** | **No*** | **3** | **Yes (negative)** | **IgM and IgG** | **No** | **Yes** |
| 3 | **M** | **85** | **Non-Hodgkin Lymphoma** | **Chemotherapy** | **R-COMP** | **Yes (cough)** | **6** | **Yes (negative)** | **Weak IgM** | **Yes** | **Yes** |
| 4 | **M** | **80** | **Non-Hodgkin Lymphoma** | **Chemotherapy** | **R-COMP** | **No*** | **1** | **Yes (negative)** | **IgM** | **No** | **Yes** |
| 5 | **F** | **64** | **Multiple Myeloma** | **Chemotherapy** | **Velcade,Thalidomide,**  **Desametazone** | **No*** | **3** | **Yes (negative)** | **IgM and IgG** | **No** | **Yes** |
| 6 | **F** | **75** | **Multiple Myeloma** | **Chemo-immunotherapy** | **Elotuzumab, Lenalidomide, Desametazone** | **No*** | **5** | **Yes (negative)** | **IgM and IgG** | **No** | **Yes** |
| 7 | **F** | **49** | **Non-Hodgkin Lymhoma** | **immunotherapy** | **Rituximab** | **No*** | **4** | **Yes (negative)** | **IgM and IgG** | **No** | **Yes** |
| 8 | **M** | **58** | **Non-Hodgkin Lymhoma** | **Chemotherapy** | **Rituximab-Bendamustine** | **No*** | **2** | **Yes (negative)** | **Weak IgM** | **No** | **Yes** |
| 9 | **M** | **49** | **Colorectal**  **Cancer** | **Chemo- and Target Therapy** | **Fluorouracile, Leucovorin, Oxaliplatino, Panitumumab** | **Yes (fever)** | **6** | **Yes (negative)** | **IgM and IgG** | **Yes** | **Yes** |
| 10 | **M** | **57** | **Gastric**  **Cancer** | **Chemotherapy** | **Fluorouracile, Leucovorin, Oxaliplatino** | **No*** | **7** | **Yes 1 Positive** | **IgM and IgG** | **Yes** | **Yes** |
| 11 | **M** | **65** | **Lung**  **Cancer** | **Chemo-immunotherapy** | **Cisplatino, Pemetrexed, Pembrolizumab** | **No*** | **3** | **Yes (negative)** | **IgM** | **No** | **Yes** |
| 12 | **M** | **61** | **Pharyngeal Cancer** | **Chemotherapy** | **Paclitaxel** | **No*** | **6** | **Yes (negative)** | **IgM** | **No** | **Yes** |
| 13 | **F** | **52** | **Breast**  **Cancer** | **Chemotherapy** | **Paclitaxel** | **No*** | **7** | **Yes (negative)** | **IgM and IgG** | **No** | **Yes** |
| 14 | **F** | **72** | **Gastric**  **Cancer** | **Immunotherapy** | **Pembrolizumab** | **No*** | **2** | **Yes (negative)** | **IgG** | **No** | **Yes** |

*No symptoms during the study period nor in the 3 months before.
